# What the papers say

**DOI:** 10.1093/jhps/hnad047

**Published:** 2023-12-30

**Authors:** Ali Bajwa

**Affiliations:** Villar Bajwa Practice, Princess Grace Hospital, London W1G6PU, United Kingdom

## Abstract

The *Journal of Hip Preservation Surgery* (*JHPS*) is not the only place where work in the field of hip preservation can be published. Although our aim is to offer the best of the best, we are continually fascinated by work, which finds its way into journals other than our own. There is much to learn from it, and so *JHPS* has selected six recent and topical subjects for those who seek a summary of what is taking place in our ever-fascinating world of hip preservation. What you see here are the mildly edited abstracts of the original articles, to give them what *JHPS* hopes is a more readable feel. If you are pushed for time, what follows should take you no more than 10 min to read. So here goes …

## ARTHROSCOPIC TREATMENT OF DEEP GLUTEAL SYNDROME AND THE APPLICATION VALUE OF HIGH-FREQUENCY ULTRASOUND

The authors from China [1] aimed to evaluate the efficacy of arthroscopic sciatic neurolysis for treating deep gluteal syndrome (DGS) and to analyse the application value of high-frequency ultrasound during perioperative period.

Between June 2020 and February 2022, 30 patients with DGS who underwent failed conservative treatment were retrospectively analysed. Lateral arthroscopic exploration of the deep gluteal space and sciatic neurolysis was performed. In addition to pelvic X-ray, lumbar disc and hip MRI, ultrasonography of the sciatic nerve was also performed in all patients. The visual analogue scale (VAS) pain score, modified Harris hip score (mHHS) and Benson symptom-rating scale were used to evaluate the clinical efficacy. The correlation between preoperative sciatic nerve ultrasound and arthroscopic findings was analysed.

They report that the median follow-up for these patients was 13 months. Preoperative ultrasonography showed precise morphological changes in 26 sciatic nerves of patients. The VAS score significantly decreased from 5.0 (4.0, 6.0) preoperatively to 0.5 (0, 1.0) postoperatively, and the mHHS significantly increased from 64.0 (57.0, 67.0) preoperatively to 95.0 (93.0, 97.0) postoperatively. The Benson symptom score was excellent in 15 cases, good in 12 cases, fair in 2 cases and poor in 1 case; thus, the score was excellent or good in 90% of the cases. Preoperative ultrasound diagnosis and intraoperative findings matched up in all cases. There were four cases of transient numbness in the posterior thigh.

The authors thus concluded that the arthroscopic sciatic neurolysis is a safe and effective treatment option for DGS patients who fail conservative treatment. Ultrasound diagnosis matched the arthroscopic findings perfectly. Preoperative Doppler ultrasound can assist surgical decision-making and guide intraoperative release.

## WHAT HAPPENS TO ENDOSCOPIC/ARTHROSCOPIC TENOTOMIES WITH ILIOPSOAS IMPINGEMENT IN THE MEDIUM-TERM? REVIEW OF A PROSPECTIVE COHORT OF 64 PATIENTS WITH A MINIMUM FOLLOW-UP OF 5 YEARS

The authors from multiple European institutions and Fancophone Arthroscopy Society [2] state that the occurrence of iliopsoas impingement (IPI) after total hip arthroplasty (THA) is a proven risk factor for negative outcomes. Endoscopic or arthroscopic tenotomies of the iliopsoas offer a surgical solution with short-term results that have already been validated in the prospective multicentre series. They carried out a review of the patients at more than 5 years of follow-up in order to assess the stability of the results over time.

Their main hypothesis was that endoscopic/arthroscopic tenotomies allow stable medium-term resolution of the painful symptoms of IPI. Their secondary hypothesis was that the medium-term survival was satisfactory.

This study is a continuation of a multicentre prospective series. Patients were contacted through multiple channels in order to obtain an Oxford score, assess for satisfaction, psoas irritation and daily pain on a VAS.

Of 64 patients in the original study, 57 were contacted. The Oxford score at the last follow-up was 40.7 ± 7.7. There was a significant difference between the Oxford scores preoperatively, at 8 months and at the last follow-up. The mean satisfaction out of 10 was 8.0 ± 2.1. They found 84% satisfaction at 5 years against 83% at 8 months. The VAS was 2.1 ± 2.3. A straight leg psoas sign was present in 19.6% (10/51) of patients at 5 years, compared to 15.6% (8/51) at 8 months. The sign disappeared in four cases, while it reappeared during the interval in six cases. The survival was 91.2% at 5 years.

The authors concluded that endoscopic/arthroscopic iliopsoas tenotomies represent a permanent medium-term solution to treat IPI after THA. The existence of a force differential or an acetabular overhang does not seem, within a certain limit, to impact the results in the medium term.

## BORDERLINE HIP DYSPLASIA IS NOT ASSOCIATED WITH SIGNIFICANT DIFFERENCES IN HIP SURVIVORSHIP OR PATIENT-REPORTED OUTCOMES FOLLOWING PRIMARY HIP ARTHROSCOPY FOR FEMOROACETABULAR IMPINGEMENT SYNDROME: A PROPENSITY-MATCHED COHORT STUDY

In this Level III study, Li *et al*. [3] compared hip survivorship and patient-reported outcomes after primary hip arthroscopy for femoroacetabular impingement syndrome (FAIS) in patients with versus without comorbid borderline hip dysplasia (BHD) at a 2-year follow-up.

They conducted a retrospective matched-cohort study involving patients who underwent primary hip arthroscopy for FAIS with a single surgeon from 2010 to 2019. BHD was defined as lateral centre-edge angle (LCEA) of 20° to 25°. Subjects with BHD were matched 1:2 to controls without BHD on age, sex, body mass index and preoperative mHHS. Alpha angle, LCEA, Tönnis angle and acetabular retroversion signs were measured on preoperative and/or postoperative hip radiographs. Patient-reported outcomes were assessed using the mHHS and the Non-Arthritic Hip Score. Hip survivorship, outcome scores and achievement of the minimum clinically important difference were compared between groups using the Mann–Whitney U test or Fisher’s exact test, as appropriate. *P* values <0.05 were considered significant.

Thirty-one BHD subjects (mean age 36.8 years, 71.0% female) and 62 controls (mean age 38.0 years, 71.0% female) were included. There were no significant intergroup differences in demographics or preoperative radiographic measurements besides LCEA and Tönnis angle. Intraoperatively, subjects with BHD were found to have significantly shorter labral tears (mean 2.6 versus 2.8 clock-face hours), but there were no significant intergroup differences in acetabular or femoral cartilage status. Postoperatively, there were no significant intergroup differences in rates of revision arthroscopy (BHD 6.5% versus control 11.3%) or conversion to THA (BHD 9.7% versus control 1.6%), in 2 year improvement of the mHHS and Non-Arthritic Hip Score or in minimum clinically important difference achievement rates.

In conclusion, the authors state that BHD is not associated with a significant difference in hip survivorship or patient-reported outcomes following primary hip arthroscopy for FAIS.

## HIP DISORDERS IN BULL RIDERS: CLINICAL OBSERVATIONS AND OUTCOMES OF ARTHROSCOPY

In this interesting study, Byrd *et al*. [4] note that the bull riders represent a microcosm of athletes in whom severe consequences of femoroacetabular impingement may challenge the limits of arthroscopic intervention. Observations of this cohort may provide meaningful insight into the treatment of other populations. All patients undergoing hip arthroscopy were prospectively assessed with an mHHS. Sixteen consecutive bull riders (21 hips) with a minimum 2-year follow-up were reported. The average age was 26 years, duration of symptoms was 33 months, and follow-up was 57 months. Arc of rotational hip motion averaged 31°. All had femoroacetabular impingement (17 combined and 4 cam impingements). Among the cam impingements, one also had dysplasia and one also had borderline dysplasia. Radiographic Tönnis grades were as follows: 2 Tönnis 1, 18 Tönnis 2 and 1 Tönnis 3. All had acetabular articular damage (14 Outerbridge grade 4, 6 Outerbridge grade 3 and 1 Outerbridge grade 1). Nine underwent microfracture. Four had accompanying femoral chondral lesions (3 grade 3 and 1 grade 4). There were 20 labral tears (14 repaired and 6 debrided). All but one (95%) improved after surgery. One bilateral case underwent conversion to resurfacing arthroplasty on one side and revision arthroscopy on the other. The average improvement was 21.3 points. Thirteen (81.25%) returned to bull riding at an average of 7 months. Among the three who did not return, each had undergone bilateral procedures. There were no complications. Femoroacetabular impingement can be a significant problem among bull riders. Limited range of motion, grade 4 articular damage and Tönnis 2 radiographic changes may not preclude successful arthroscopic treatment, but advanced bilateral disease may be too much even for these hardened athletes.

## A SCOPING REVIEW: SEXUAL ACTIVITY AND FUNCTIONING BEFORE AND AFTER SURGERY FOR FEMOROACETABULAR IMPINGEMENT (FAI), LABRAL TEARS, AND HIP DYSPLASIA

The authors from FL and TX, USA [5], note that there is limited information on sexual activity and functioning for patients with hip abnormalities, specifically femoroacetabular impingement (FAI), labral tears and hip dysplasia before and after surgical interventions.

The aim of their review was to synthesize the existing literature on sexual activity and functioning for patients with FAI, labral tears and/or hip dysplasia before and after their respective surgeries.

They performed a rigorous, comprehensive search on multiple databases including PubMed, EMBASE, CINAHL and Web of Science. Subject headings and a search string of key terms including Medical Subject Headings were used systematically to search these databases. The reference list was reviewed with an additional reviewer to reduce bias.

They found a total of 726 articles during their search, which were narrowed down to 22 articles that included at least one hip abnormality in relation to sexual functioning, sexual pain or sexual activity. FAI, labral tears and hip dysplasia can affect sexual activity, functioning and positioning, and the corrective surgery generally improves these metrics. The surgery improved vulvodynia, clitorodynia and scrotal pain symptoms for some patients, though arthroscopy resulted in some instances of temporary pudendal nerve dysfunction.

The authors concluded that this review may serve as an important resource for surgeons, healthcare providers, researchers, physical therapists and patients to understand the relationship between hips and sexual functioning and to bridge the gaps among the disciplines of orthopaedics, pelvic floor physiology and sexual health. Hip anatomy impacts sexual activity, functioning and positioning as well as vulvodynia and scrotal pain symptoms for some patients, and a comprehensive hip evaluation by a qualified hip specialist should be considered for patients with such complaints.

## OUTCOMES OF HIP ARTHROSCOPY IN THE SETTING OF CONCOMITANT SYMPTOMATIC LUMBOSACRAL SPINE PATHOLOGY: A MATCHED CONTROL STUDY WITH MINIMUM 24-MONTH FOLLOW-UP

The authors from multiple centres in the USA [6] note that the overlapping biomechanical relationship between the lumbosacral spine and pelvis poses unique challenges to patients with concomitant pathologies, limiting spinopelvic range of motion. They set out to assess the influence of concomitant, symptomatic lumbosacral spine pathology on patient-reported outcome measures (PROMs) after hip arthroscopy for the treatment of FAI and symptomatic labral tears in this Level-3 cohort study.

A retrospective query of prospectively collected data, which identified patients aged ≥18 years with a minimum 24-month follow-up who underwent hip arthroscopy by a single surgeon for the treatment of symptomatic labral tears secondary to FAI, was created. Patients were stratified into cohorts based on the presence [hip-spine (HS)] or absence [matched control (MC)] of symptomatic lumbosacral spine pathology. Inclusion within the HS cohort required confirmation of lower back pain/symptoms on preoperative surveys plus a diagnosis of lumbosacral spine pathology verified by radiology reports and correlating clinical documentation. Patients with previous spine surgery were excluded. PROMs were compared between groups, along with rates of achieving minimal clinically important difference (MCID) thresholds, Patient Acceptable Symptom State (PASS) thresholds, revision arthroscopy, and conversion to THA.

A total of 70 patients with lumbosacral pathology were coarsened exact matched to 87 control patients without spinal pathology. The HS cohort had preoperative baseline scores that were significantly worse for nearly all PROMs. Follow-up at 3, 6, 12 and 24 months displayed similar trends, with the HS cohort demonstrating significantly worse scores for most collected outcomes. However, at every time point, HS and MC patients exhibited similar magnitudes of improvement across all PROM and pain metrics. Furthermore, while significantly fewer HS patients achieved PASS for nearly all PROMs at 12- and 24-month follow-ups, MCID thresholds were reached at similar or greater rates across all PROMs relative to the MC cohort. Finally, there were no significant differences in rates of revision or THA between cohorts at maximum available follow-up.

The authors concluded that after hip arthroscopy to address labral tears in the setting of FAI, patients with symptomatic lumbosacral pathologies and no history of spine surgery were found to exhibit inferior pre- and postoperative PROMs but achieved statistically similar clinical benefit and rates of PROM improvement through 24-month follow-up compared with the MC cohort with isolated hip disease. These findings aid in providing a realistic recovery timeline and evidence that coexisting hip and spine disorders are not a contraindication for arthroscopic hip preservation surgery.
